# Characteristics and Prognostic Analysis in Diving-Induced Ear Trauma and Sudden Hearing Loss

**DOI:** 10.3390/jcm15103870

**Published:** 2026-05-18

**Authors:** Ting-Chun Yi, Tsu-Hsuan Weng, Hsin-Chien Chen

**Affiliations:** 1 Department of Otolaryngology-Head and Neck Surgery, Tri-Service General Hospital, National Defense Medical University, Taipei 11490, Taiwan; tingy199519@gmail.com; 2 Department of Medical Research, Tri-Service General Hospital, National Defense Medical University, Taipei 11490, Taiwan; a0984295524@gmail.com; 3 Department of Otolaryngology-Head and Neck Surgery, School of Medicine, College of Medicine, National Defense Medical University, Taipei 11490, Taiwan

**Keywords:** scuba diving, decompression sickness, hyperbaric oxygen, hearing loss, ear, barotrauma, vertigo, tinnitus

## Abstract

**Background/Objectives**: Diving exposure can cause auditory injury involving both the middle and inner ear structures. Inner ear barotrauma (IEB) and inner ear decompression sickness (IEDCS) are the major inner ear disorders and frequently present with auditory and vestibular symptoms. This study examined how diving characteristics relate to patterns of auditory trauma. **Methods**: A retrospective chart review of 30 patients, with 36 affected ears, was performed. Diving depth, clinical manifestations, and treatment responses were analyzed to identify factors influencing related prognosis. **Results**: Diving depth was an important factor associated with symptom severity and the type of injury. Dives deeper than 30 m of seawater were linked to a higher incidence of sudden sensorineural hearing loss and vertigo. In contrast, transient symptoms with minimal objective abnormalities were typically observed in shallow dives. Patients with concomitant decompression sickness (DCS) showed poorer auditory and vestibular recovery following hyperbaric oxygen therapy, while those without DCS showed better hearing improvement. Vertigo was observed in 80% of IEB cases and 66.7% of IEDCS cases. Hearing recovery appeared to be more frequently observed in cases presenting with middle ear symptoms, suggesting a relatively favorable prognosis for IEB compared with IEDCS. **Conclusions**: The findings suggest potential associations between diving depth and DCS, and its involvement may play a role in the severity and prognosis of diving-related inner ear injury. IEB appeared to be associated with more favorable auditory outcomes compared with IEDCS; however, this observation should be interpreted with caution due to potential diagnostic uncertainty. Given the descriptive nature of the study, further studies with larger cohorts are needed to refine prognostic indicators and optimize management strategies.

## 1. Introduction

Self-contained underwater breathing apparatus diving (SCUBA diving) is a popular recreational activity; however, rapid ambient pressure changes, ascent rate, breathing gas composition, and repeated exposure may predispose individuals to auditory injury involving the outer, middle, and inner ear [1,2,3]. Decompression sickness (DCS), historically referred to as caisson disease, is a well-recognized condition caused by inert gas bubble formation during rapid decompression and may involve multiple organ systems, including the inner ear. These exposure variables may affect both middle and inner ear structures through barotrauma or inert gas bubble formation [2,4]. Inner ear barotrauma (IEB) and inner ear decompression sickness (IEDCS) represent two distinct forms of inner ear injuries with similar clinical manifestations [5]. Most affected individuals present with sudden sensorineural hearing loss accompanied by tinnitus and vertigo [6]. According to the literature, IEB is commonly associated with middle ear findings such as hemorrhage, tympanic membrane (TM) perforation, and otorrhea, whereas IEDCS is more often accompanied by musculoskeletal pain, as well as cardiopulmonary and neurological symptoms. Cardiopulmonary manifestations may include dyspnea, chest pain, cough, tachypnea, and in more severe cases, cyanosis or hemodynamic instability, reflecting pulmonary involvement. Neurological symptoms are also common and may range from dizziness, headache, and paresthesia to more severe presentations such as motor weakness, ataxia, visual disturbances, cognitive impairment, or altered consciousness, depending on the extent and location of gas emboli affecting the central or peripheral nervous system [7,8]. Currently, no definitive clinical diagnostic method exists to reliably differentiate between the two conditions [5]. Consequently, without thorough and detailed history-taking, establishing an accurate diagnosis based solely on clinical presentation remains challenging and may lead to delays in initiating appropriate treatment.

Previous studies investigating the prognosis of idiopathic sudden hearing loss have consistently reported a significant therapeutic benefit from hyperbaric oxygen therapy (HBOT), which has been reported to provide additional benefits when used as an adjunct to pharmacological treatment, particularly early steroid treatment in the initial stages [9,10,11]. Initiating HBOT treatment within 14 days of symptom onset is considered critical for optimizing outcomes [12,13]. HBOT has also demonstrated favorable efficacy in cases of post-diving sudden hearing loss; however, variability in recovery suggests that treatment response alone cannot fully explain prognosis [5,14,15,16,17]. These discrepancies highlight the need for further investigation. Several clinical features, including diving depth, presence of vertigo, systemic decompression symptoms, and timing of treatment initiation, have been suggested as potential prognostic determinants of hearing recovery. However, existing evidence remains limited and inconsistent. Most previous investigations primarily emphasized treatment efficacy rather than the correlation between exposure variables and prognostic outcomes. Consequently, the relationship between diving exposure characteristics and auditory prognosis remains incompletely understood.

The present study aims to examine the prognostic risk factors associated with traumatic hearing loss occurring during diving activities. Specifically, it seeks to analyze the correlation between diving exposure and hearing impairment, compare the severity and patterns of hearing loss with various diving modes, and identify contributing factors that may predispose individuals to barotrauma or decompression sickness following diving-related sudden hearing loss.

## 2. Materials and Methods

**Figure 1:**
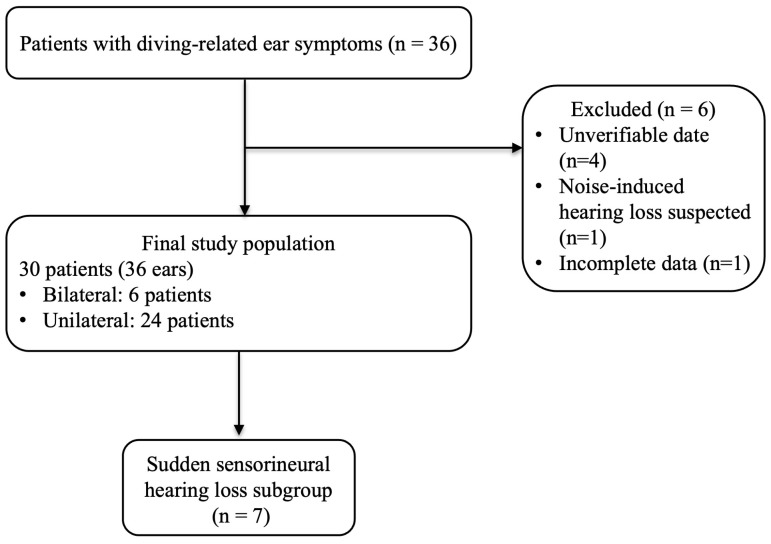
Flowchart of patient selection.

This study included patients who presented to our hospital with auditory or vestibular symptoms following diving between 1 January 2010, and 30 November 2023, and who underwent comprehensive audiological evaluation. This study protocol was approved by the Institutional Review Board of Tri-Service General Hospital, Taipei, Taiwan (IRB no. B202405094; approval date: 2 May 2024). A total of 36 patients were initially identified. After excluding four patients with unverifiable diving dates, one patient with suspected noise-induced hearing loss, and one patient with incomplete examination data, 30 patients were enrolled. Overall, 6 patients exhibited bilateral ear involvement and 24 had unilateral ear involvement, yielding a total of 36 ears for analysis. Ear-level analysis was performed to capture ear-specific clinical characteristics, as auditory findings may differ between ears in the same patient. The patient selection process is illustrated in Figure 1.

Given the limited sample size, the study was designed as a descriptive analysis. Therefore, the results are presented primarily using descriptive statistics without formal inferential testing. The 36 ears were classified according to three frameworks. First, ears were grouped by diving depth: ≤10 m of seawater (msw; Group A), 11–20 msw (Group B), 21–30 msw (Group C), and 31–40 msw (Group D). Second, ears were categorized by diagnosis into five groups: normal hearing with intact TM (NH + intact TM), middle ear barotrauma with TM perforation (MEB + perforated TM), middle ear barotrauma with intact TM (MEB + intact TM), IEB, and IEDCS. The IEB and IEDCS classification was based on clinical assessment, including diving profile (depth and ascent), timing of symptom onset, presence of middle ear findings, and associated systemic decompression-related symptoms. This approach was adapted from previously published diagnostic considerations, given the absence of a definitive diagnostic standard.

Finally, in the subgroup with sudden hearing loss, seven patients who met the diagnostic criteria for sudden sensorineural hearing loss (SSNHL) were identified and analyzed separately. The following variables were collected: demographic data (age, sex), diving-related parameters (diving depth, interval from symptom onset to treatment initiation), clinical characteristics (affected ear, chief complaints, and diagnosis), and audiological and vestibular assessments, including pure-tone audiometry (PTA), videonystagmography (VNG), and caloric testing. Reported auditory and vestibular symptoms included tinnitus, vertigo, aural fullness, otalgia, and otorrhea. Additional decompression-related symptoms, such as headaches, muscle weakness, joint pain, and cardiopulmonary dysfunction, were also documented. Treatment-related variables comprised HBOT protocol, number of HBOT sessions, cumulative HBOT duration, and number of intratympanic steroid injections (ITSIs). ITSIs were administered with a dosage of 0.8 mL using RINDERON^®^ INJECTION 4 mg/mL (Betamethasone) (Taiwan Shionogi & Co., Ltd., Taipei, Taiwan). The interval between injections typically ranged from 2 to 5 days. Treatment allocation (HBOT, ITSI, or combined therapy) was not randomized and was determined based on the clinical severity of hearing loss, presence of vestibular or systemic decompression-related symptoms, physician judgment, availability of hyperbaric facilities, and patient preference.

Prognostic outcomes included hearing recovery measured in decibels (dB), the degree of hearing recovery, and the presence of residual vestibular symptoms. The degree of prognosis was defined as no recovery (PTA improvement ≤ 10 dB HL), partial recovery (PTA improvement > 10 dB HL with a residual interaural difference > 10 dB HL), or total recovery (PTA improvement > 10 dB HL with a residual interaural difference ≤ 10 dB HL or a final hearing threshold ≤ 20 dB HL).

The authors acknowledge the use of ChatGPT (GPT-5.5, OpenAI, San Francisco, CA, USA) for language editing assistance in the preparation of this manuscript. The authors take full responsibility for the content.

### 2.1. Audiological Assessment

Audiological evaluations were performed using a calibrated clinical audiometer, the GSI AudioStar Pro (Grason-Stadler, Eden Prairie, MN, USA). PTA was conducted in a sound-treated booth by certified audiologists. Air conduction thresholds were measured at frequencies ranging from 250 to 8000 Hz, and bone conduction thresholds were assessed from 500 to 4000 Hz, in accordance with standard clinical protocols. Appropriate masking was applied when required, following established audiological guidelines.

Tympanometry was performed to assess middle ear function, and tympanic membrane status was confirmed through otoscopic examination. The PTA was calculated based on thresholds at 500, 1000, 2000, and 4000 Hz.

### 2.2. Vestibular Assessment

Vestibular function was evaluated using VNG and caloric testing. The presence of spontaneous nystagmus and canal paresis was recorded. Caloric weakness was interpreted according to standard clinical criteria (CP value ≥25%). Additional vestibular symptoms, including vertigo and imbalance, were documented based on patient-reported outcomes.

### 2.3. Statistical Analysis

Statistical analyses were performed using IBM SPSS Statistics (version 32; IBM Corp., Armonk, NY, USA). The distribution of continuous variables was assessed using the Shapiro–Wilk test. A *p*-value ≥ 0.05 was considered to indicate no significant deviation from normality. Continuous variables are presented as mean ± standard deviation (SD), whereas categorical variables are presented as counts and percentages. In the SSNHL subgroup, both mean ± SD and median with interquartile range (IQR) are presented to provide a more comprehensive description of the data distribution. Given the limited sample size and subgroup heterogeneity, inferential statistical analyses were not performed, and the results are presented descriptively.

## 3. Results

The study included 30 patients with a mean age of 37.36 ± 9.92 years, comprising 17 males and 13 females. The mean hearing threshold of the 36 affected ears was 28.34 ± 23.61 dB HL. The distribution of age was consistent with normality (*p* = 0.449), whereas the distribution of PTA values (*p* < 0.001) and diving depth (*p* = 0.037) deviated from normality. Given the non-normal distribution and limited sample size, the data were interpreted descriptively without formal inferential statistical comparisons.

### 3.1. Characteristics and Analysis of Different Diving Depth Groups

**Figure 2:**
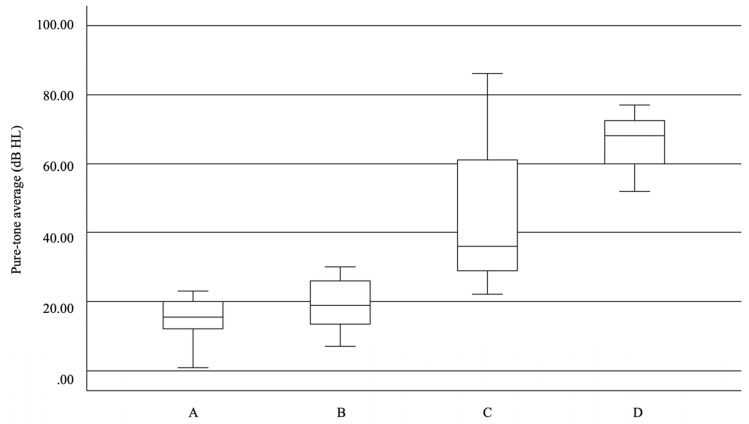
Distribution of pure-tone average values across different diving depth groups. The box represents the interquartile range, with the central line indicating the median. Whiskers indicate the range of values. Groups A, B, C, and D represent diving depths of 0–10, 11–20, 21–30, and 31–40 msw, respectively.

The characteristics of the four diving depth groups are summarized in Table 1. Mean PTA thresholds increased progressively with depth, from 15.5 dB HL in Group A (≤10 msw) to 66.25 dB HL in Group D (31–40 msw), indicating greater hearing loss at deeper exposures (Figure 2). The distribution of diagnoses also varied by depth. IEDCS was observed only in the 31–40 msw group, whereas normal hearing with an intact TM appeared more common at depths ≤ 20 msw. Vertigo appeared to be more frequently reported in the deepest group, while otalgia was limited to depths ≤ 20 msw. Tinnitus was observed across multiple depth categories. Notably, individuals in the ≤10 msw group more often presented with subjective symptoms without objective clinical findings, suggesting that auditory symptoms following shallow dives may be transient and not associated with structural ear injury.

### 3.2. Analysis of Different Diagnostic Categories

**Figure 3:**
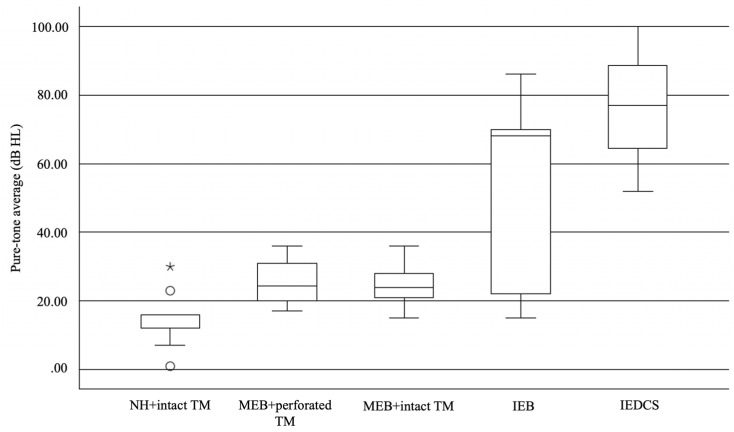
Distribution of pure-tone average values across different diagnostic groups. The box represents the interquartile range, with the central line indicating the median. Whiskers indicate the range of values. Circles indicate outliers, and asterisks indicate extreme outliers.

Characteristics of the five diagnostic categories—normal hearing with intact TM (NH + intact TM), middle ear barotrauma with perforated TM (MEB + perforated TM), middle ear barotrauma with intact TM (MEB + intact TM), IEB, and IEDCS—are summarized descriptively in Table 2. Mean PTA thresholds varied across diagnostic groups (Figure 3). Greater hearing loss appeared to be observed in patients diagnosed with IEDCS and IEB compared with those with middle ear barotrauma or normal hearing. Vertigo was reported more frequently in patients with IEDCS and IEB than in other diagnostic groups.

### 3.3. Descriptive Analysis of Patients with SSNHL

The results for patients with SSNHL are presented in Table 3. Given the small number of cases, these findings should be considered exploratory and descriptive. Among the seven patients with SSNHL, five patients were male and two were female, with ages ranging from 21 to 62 years (mean age = 36.29 ± 14.56 years; median = 32.00; IQR: 26.00–43.50). The most frequently reported symptoms were vertigo (*n* = 5, 71.4%), tinnitus (*n* = 4, 57.1%), and aural fullness (*n* = 3, 42.9%). Additional symptoms, including otalgia, otorrhea, headaches, muscle weakness, joint pain, and cardiopulmonary dysfunction, were each reported by one patient. All seven patients were scuba divers, with a mean diving depth of 29.83 ± 8.75 msw (median = 31.00; IQR: 20.00–37.00). Hearing loss was right-sided in three patients and left-sided in four patients, with a mean PTA threshold of 68.39 ± 25.06 dB HL (median = 71.25; IQR: 60.63–81.88). Vestibular function testing was performed in four patients, two of whom (50%) demonstrated unilateral caloric weakness (CP value = 30% and 74%) and the others (50%) demonstrated bilateral caloric weakness (CP value = 50% and 10%); one patient (25%) also exhibited eight degrees of spontaneous nystagmus on VNG. The mean interval from symptom onset to treatment initiation was 120 ± 45.96 h (median = 120.00; IQR: 96.00–156.00). In addition to pharmacological therapy, two patients received combined HBOT and ITSIs, three patients received HBOT alone, and two received ITSIs alone. ITSIs were administered with a dosage of 0.8 mL using RINDERON^®^ INJECTION 4 mg/mL (Betamethasone) (Taiwan Shionogi & Co., Ltd., Taipei, Taiwan). The treatment intervals ranged from 2 to 5 days. Among the five patients treated with HBOT, three underwent standard HBOT (253 kPa [2.5 ATA] for 100 min), one received treatment according to the United States Navy Diving Manual, Revision 5 (USN-5; 284 kPa [2.8 ATA] for 138 min), and one underwent a combination of both protocols. The mean hearing improvement was 21.96 ± 28.44 dB HL (median = 10.00; IQR: 4.38–36.88). Among the three patients diagnosed with IEDCS, two presented with additional decompression-related symptoms, including headaches, musculoskeletal pain, and cardiopulmonary dysfunction; all these symptoms resolved following HBOT. Overall, complete hearing recovery was observed in two patients (28.5%), partial recovery in one patient (14.3%), and no recovery in four patients (57.1%). Hearing recovery categories were defined according to changes in PTA and interaural hearing thresholds, as detailed in Table 3. Persistent vestibular symptoms were reported in four patients (57.1%), including individuals with both complete and absent hearing recovery. Among patients who received HBOT, hearing recovery was achieved in three cases (60%), including two with complete and one with partial recovery.

## 4. Discussion

IEB and IEDCS may be differentiated based on several clinical characteristics, including diving profile, breathing gas composition, timing of symptom onset, and associated manifestations. IEB may occur during either descent or ascent, whereas IEDCS typically occurs during ascent, with symptoms emerging minutes to hours after surfacing. Although both conditions may present with sudden hearing loss accompanied by tinnitus and vertigo, IEDCS appears more frequently associated with vertigo (91.7%) and systemic decompression-related symptoms, whereas IEB is more often accompanied by middle ear findings [5]. In the present study, patients presenting with sudden sensorineural hearing loss accompanied by middle ear symptoms and without systemic decompression-related manifestations were more likely to be classified as having IEB. However, given the overlap in clinical presentations and the lack of a definitive diagnostic standard, misclassification between IEB and IEDCS cannot be entirely excluded.

In the present study, variations in hearing loss severity, diagnostic distribution, and symptom patterns were observed across different diving depths. Greater hearing loss and a higher proportion of IEB and IEDCS were noted in deeper diving groups (31–40 msw), which also showed a higher prevalence of vertigo. These findings suggest a potential association between diving depth and the severity of auditory and vestibular manifestations. In contrast, shallow dives (0–10 msw) were more frequently associated with subjective symptoms without objective abnormalities, possibly reflecting lower pressure gradients and a reduced risk of structural injury. Tinnitus was observed in both shallow and deep dives, whereas otalgia was mainly reported in divers exposed to depths ≤ 20 msw (Table 1, Figure 2).

When comparing diagnostic categories, patients classified as IEB and IEDCS appeared to demonstrate greater hearing loss than those with middle ear barotrauma or normal findings (Figure 3). Vertigo was also more frequently observed in IEB and IEDCS groups, suggesting that vestibular involvement may be a useful clinical indicator of inner ear pathology (Table 2). However, these observations should be interpreted cautiously given the descriptive nature of the study and the limited sample size.

Decompression sickness results from inert gas bubble formation during rapid reductions in ambient pressure and may involve multiple organ systems [18]. Inner ear involvement has been reported in a substantial proportion of Type II decompression sickness cases, with a considerable number of patients experiencing persistent auditory or vestibular deficits despite treatment [2,5,19,20]. Early recompression and HBOT remain the cornerstone of management, although treatment protocols vary across institutions and clinical settings [13]. In Taiwan, USN-5A (608 kPa [6.0 ATA] with 154 min) and USN-6A (608 kPa [6.0 ATA] with 319 min) protocols are commonly employed, followed by standard HBOT for residual symptoms, a strategy also applicable to sudden sensorineural hearing loss.

In the present cohort of patients with SSNHL, both median with IQR and mean ± SD were reported to provide a more comprehensive description of the data. The differences between these measures suggest variability in the distribution, and therefore the findings should be interpreted with caution. The early initiation of HBOT appeared to be associated with improved hearing outcomes, consistent with previous reports [7,21,22]. However, extended HBOT duration (>900 min) did not uniformly predict favorable prognosis, and some patients continued to experience persistent vestibular symptoms despite treatment. These findings suggest that factors such as diving depth, treatment delay, intratympanic steroid use, and the presence of systemic decompression-related symptoms may influence outcomes. Nevertheless, given the small sample size, these observations should be considered exploratory and hypothesis-generating rather than conclusive.

Patients classified as IEDCS often demonstrated improvement in systemic symptoms following HBOT, while auditory and vestibular recovery appeared more limited. In contrast, cases with middle ear findings suggestive of IEB, such as aural fullness, otalgia, and otorrhea, were more frequently associated with hearing recovery. This pattern may indicate differing underlying pathophysiological mechanisms; however, the observed differences should be interpreted with caution due to potential diagnostic uncertainty and treatment selection bias.

Regardless of the pressure level, 203 kPa (2.5 ATA) or 253 kPa (2.8 ATA), HBOT appeared to be beneficial for hearing recovery, particularly in patients without systemic decompression-related symptoms, although comparisons between treatment modalities are limited by non-randomized allocation and potential selection bias. Vestibular symptoms, in contrast, appeared less responsive to treatment. Managing persistent vestibular symptoms may include the short-term use of vestibular suppressants and vestibular rehabilitation therapy (VRT), although treatment strategies should be individualized based on clinical presentation and disease severity. An accurate differentiation between IEB and IEDCS is therefore essential and should incorporate diving profile, gas composition, symptom onset timing, and the presence of vertigo or systemic decompression symptoms. In cases where sudden hearing loss after diving is accompanied by systemic decompression manifestations, aggressive HBOT and early vestibular rehabilitation should be considered to optimize outcomes [23].

Perilymphatic fistula (PLF) should be considered in the differential diagnosis of acute audiovestibular symptoms after diving. However, diagnosis is challenging, and exploratory tympanotomy is generally reserved for cases with high clinical suspicion. In the present study, due to the lack of suggestive features, none of the patients underwent surgical exploration, although occult PLF could not be ruled out. The use of HBOT in this condition is controversial, as increased pressure may theoretically worsen perilymph leakage. Nevertheless, HBOT is commonly applied in cases of diagnostic uncertainty. In the present study, no clinical deterioration attributable to HBOT was observed.

This study has several limitations. First, treatment allocation was not randomized, and the choice of HBOT, ITSIs, or combined therapy was influenced by clinical severity, the presence of systemic symptoms, physician judgment, and patient preference, which may introduce selection bias. Second, each ear was analyzed as an independent unit, despite some bilateral cases, which may introduce within-subject correlation bias. Third, the sample size was small and heterogeneous, and subgroup analyses, particularly for SSNHL, should be considered exploratory with limited generalizability. Fourth, inner ear and vestibular assessment was limited to PTA and caloric testing, and additional objective measures such as otoacoustic emissions (OAEs), video head impulse testing (vHIT), and vestibular evoked myogenic potentials (VEMPs) were not available. Finally, the diagnostic classification of IEB and IEDCS relied on clinical assessment, which may introduce some diagnostic uncertainty. Given the overlap in clinical presentations and the lack of a definitive diagnostic standard, misclassification between IEB and IEDCS cannot be entirely excluded.

## 5. Conclusions

This study analyzed 36 ears to describe diving-related auditory injury and HBOT prognosis. Diving depth appeared to be associated with differences in symptom severity and the type of injury, with shallow dives more often presenting with transient auditory symptoms and deeper dives showing a higher occurrence of sudden hearing loss with vertigo and persistent vestibular symptoms. Treatment delay, number of ITSIs, and the presence of systemic decompression-related symptoms were observed as potential factors related to prognosis. Hearing recovery appeared more frequently in patients with inner ear barotrauma or without decompression-related symptoms, whereas treatment outcomes were less favorable when systemic decompression-related symptoms were present. Vestibular symptoms appeared less responsive to treatment. Given the descriptive design, small sample size, and subgroup heterogeneity, these findings should be considered hypothesis-generating and cannot establish causal relationships. Further large-scale, prospective, and multicenter studies are required to validate these observations and clarify diagnostic and therapeutic implications.

## Figures and Tables

**Table 1:** Characteristics and analysis of different diving depth groups.

Diving Depth		Total	A (0–10 msw)	B (11–20 msw)	C (21–30 msw)	D (31–40 msw)
Variables		*n* (%)	*n* (%)	*n* (%)	*n* (%)	*n* (%)
Total		29	10	12	4	3
PTA (dB HL, mean ± SD)		28.34 ± 23.61	15.50 ± 6.38	23.35 ± 16.33	45.31 ± 28.05	66.25 ± 12.69
Age (years, mean ± SD)		37.36 ± 9.92	38.20 ± 11.26	32.67 ± 7.13	41.75 ± 12.30	49.67 ± 11.59
Gender	Male	14 (48.3%)	5 (50.0%)	5 (41.7%)	1 (25.0%)	3 (100.0%)
	Female	15 (51.7%)	5 (50.0%)	7 (58.3%)	3 (75.0%)	0 (0.0%)
Affected ear	Right	18 (62.1%)	7 (70.0%)	7 (58.3%)	2 (50.0%)	2 (66.7%)
	Left	11 (37.9%)	3 (30.0%)	5 (41.7%)	2 (50.0%)	1 (33.3%)
Diagnosis	NH + intact TM	11 (37.9%)	6 (60.0%)	5 (41.7%)	0 (0.0%)	0 (0.0%)
	MEB + perforated TM	3 (10.3%)	1 (10.0%)	1 (8.3%)	1 (25.0%)	0 (0.0%)
	MEB + intact TM	8 (27.6%)	2 (20.0%)	4 (33.3%)	2 (50.0%)	0 (0.0%)
	IEB	5 (17.3%)	1 (10.0%)	2 (16.7%)	1 (25.0%)	1 (33.3%)
	IEDCS	2 (6.9%)	0 (0.0%)	0 (0.0%)	0 (0.0%)	2 (66.7%)
Tinnitus	Without	17 (58.6%)	2 (20.0%)	11 (91.7%)	3 (75.0%)	1 (33.3%)
	With	12 (41.4%)	8 (80.0%)	1 (8.3%)	1 (25.0%)	2 (66.7%)
Vertigo	Without	19 (65.5%)	9 (90.0%)	6 (50.0%)	4 (100.0%)	0 (0.0%)
	With	10 (34.5%)	1 (10.0%)	6 (50.0%)	0 (0.0%)	3 (100.0%)
Aural fullness	Without	10 (34.5%)	3 (30.0%)	5 (41.7%)	1 (25.0%)	1 (33.3%)
	With	19 (65.5%)	7 (70.0%)	7 (58.3%)	3 (75.0%)	2 (66.7%)
Otalgia	Without	21 (72.4%)	9 (90.0%)	5 (41.7%)	4 (100.0%)	3 (100.0%)
	With	8 (27.6%)	1 (10.0%)	7 (58.3%)	0 (0.0%)	0 (0.0%)

Diving depth information was available for 29 ears. Patient-level variables (age and gender) were repeated for bilateral cases. Abbreviations: dB HL = decibel hearing level; NH = normal hearing; MEB = middle ear barotrauma; IEB = inner ear barotrauma; IEDCS = inner ear decompression sickness; TM = tympanic membrane; PTA = pure-tone average.

**Table 2:** Characteristics and analysis of different diagnostic categories.

Diagnosis		Total	NH + Intact TM	MEB + Perforated TM	MEB + Intact TM	IEB	IEDCS
Variables		*n* (%)	*n* (%)	*n* (%)	*n* (%)	*n* (%)	*n* (%)
Total		36	16	4	8	5	3
PTA (dB HL, mean ± SD)		28.34 ± 23.61	14.06 ± 6.28	25.94 ± 7.80	24.88 ± 6.47	52.50 ± 31.68	76.67 ± 23.76
Age (years, mean ± SD)		37.36 ± 9.92	35.25 ± 7.88	41.50 ± 7.14	39.63 ± 11.06	31.20 ± 9.81	47.33 ± 15.01
Gender	Male	19 (52.8%)	7 (43.8%)	2 (50.0%)	4 (50.0%)	3 (60.0%)	3 (100.0%)
	Female	17 (47.2%)	9 (56.2%)	2 (50.0%)	4 (50.0%)	2 (40.0%)	0 (0.0%)
Affected ear	R	21 (58.3%)	9 (56.2%)	3 (75.0%)	5 (62.5%)	3 (60.0%)	1 (33.3%)
	L	15 (41.7%)	7 (43.8%)	1 (25.0%)	3 (37.5%)	2 (40.0%)	2 (66.7%)
Tinnitus	Without	21 (58.3%)	10 (62.5%)	2 (50.0%)	6 (75.0%)	2 (40.0%)	1 (33.3%)
	With	15 (41.7%)	6 (37.5%)	2 (50.0%)	2 (25.0%)	3 (60.0%)	2 (66.7%)
Vertigo	Without	26 (72.2%)	12 (75.0%)	4 (100.0%)	8 (100.0%)	1 (20.0%)	1 (33.3%)
	With	10 (27.8%)	4 (25.0%)	0 (0.0%)	0 (0.0%)	4 (80.0%)	2 (66.7%)
Aural fullness	Without	13 (36.1%)	4 (25.0%)	2 (50.0%)	1 (12.5%)	4 (80.0%)	2 (66.7%)
	With	23 (63.9%)	12 (75.0%)	2 (50.0%)	7 (87.5%)	1 (20.0%)	1 (33.3%)
Otalgia	Without	27 (75.0%)	12 (75.0%)	2 (50.0%)	6 (75.0%)	4 (80.0%)	3 (100.0%)
	With	9 (25.0%)	4 (25.0%)	2 (50.0%)	2 (25.0%)	1 (20.0%)	0 (0.0%)

Analyses were performed per affected ear (*n* = 36). Patient-level variables (age and gender) were repeated for bilateral cases. Abbreviations: dB HL = decibel hearing level; NH = normal hearing; IEB = inner ear barotrauma; IEDCS = inner ear decompression sickness; MEB = middle ear barotrauma; PTA = pure-tone average; TM = tympanic membrane.

**Table 3:** Individual clinical characteristics and treatment outcomes of patients with sudden hearing loss after diving.

No.		1	2	3	4	5	6	7	Mean ± SD	Median (IQR)	*n* (%)
Basic information	Gender	M	M	M	F	M	F	M	/	/	Male = 5 (71.4%) Female = 2 (28.6%)
	Age	39	32	27	21	62	25	48	36.29 ± 14.56	32.00 (26.00–43.50)	/
	msw	40	/	27	20	35	20	37	29.83 ± 8.75	31.00 (20.00–37.00)	/
	Delay (hours)	48	168	72	120	144	120	168	120 ± 45.96	120.00 (96.00–156.00)	/
Examination results	Ear	R	L	L	L	L	R	R	/	/	R = 3 (42.9%) L = 4 (57.1%)
	PTA	68.8	100	86.3	22.5	77.5	71.3	52.5	68.39 ± 25.06	71.25 (60.63–81.88)	/
	VNG	–	/	–	–	/	/	+	/	/	Normal = 3 (75%) With spontaneous nystagmus = 1 (25%)
	Caloric test	BW	/	UW	BW	/	/	UW	/	/	UW = 2 (50%) BW = 2 (50%)
	CP value	50%	/	30%	10%	/	/	74%	/	/	/
Symptom	Tinnitus	+	+	–	+	+	–	–	/	/	With = 4 (57.1%) Without = 3 (42.9%)
	Vertigo	+	–	–	+	+	+	+	/	/	With = 5 (71.4%) Without = 2 (28.6%)
	Aural fullness	+	–	+	–	–	–	+	/	/	With = 3 (42.9%) Without = 4 (57.1%)
	Otalgia	–	–	–	–	–	+	–	/	/	With = 1 (14.3%) Without = 6 (85.7%)
	Otorrhea	–	–	–	–	–	+	–	/	/	With = 1 (14.3%) Without = 6 (85.7%)
	Headache	–	–	–	–	+	–	–	/	/	With = 1 (14.3%) Without = 6 (85.7%)
	Muscle weakness	–	–	–	–	+	–	–	/	/	With = 1 (14.3%) Without = 6 (85.7%)
	Joint pain	–	–	–	–	–	–	+	/	/	With = 1 (14.3%) Without = 6 (85.7%)
	Cardiopulmonary dysfunction	–	–	–	–	–	–	+	/	/	With = 1 (14.3%) Without = 6 (85.7%)
HBOT	kPa	284 + 253	/	253	/	253	284	253	/	/	/
	Times	1 + 3	0	20	0	12	3	11	/	/	/
	Minutes	138 + 300	0	2000	0	1200	414	1100	/	/	/
ITSI	Times	0	4	10	5	3	0	0	/	/	/
	Dosages (Rinderon 4 mg/mL)	/	4	4	4	4	/	/	/	/	/
	Intervals (days)	/	2–3	3–5	3–5	2–4	/	/	/	/	/
Prognosis	dB HL	15	5	66.3	–5	3.75	58.8	10	21.96 ± 28.44	10.00 (4.38–36.88)	/
	Degree	Partial	No	Total	No	No	Total	No	/	/	No recovery = 4 (57.1%) Partial recovery = 1 (14.3%) Total recovery = 2 (28.6%)
	Vestibular symptoms	+	/	/	–	+	+	+	/	/	With = 4 (80%) Without = 1 (20%)

The degree of prognosis was defined as no recovery (PTA improvement ≤ 10 dB HL), partial recovery (PTA improvement > 10 dB HL with a residual interaural difference > 10 dB HL), or total recovery (PTA improvement > 10 dB HL with a residual interaural difference ≤ 10 dB HL or a final hearing threshold ≤ 20 dB HL). Abbreviations: dB HL = decibel hearing level; HBOT = hyperbaric oxygen therapy; ITSI = intratympanic steroid injection; kPa = kilopascals; msw = meters of seawater; PTA = pure-tone average; delay = delay onset of treatment; UW = unilateral weakness; BW = bilateral weakness; CP value = canal paresis value; Vestibular symptoms = residual vestibular symptoms.

## Data Availability

The datasets used and/or analyzed during the current study are available from the corresponding author upon reasonable request.

## References

[1] Evens R.A., Bardsley B., Manchaiah V.K.C. (2012). Auditory complaints in scuba divers: An overview. Indian J. Otolaryngol. Head Neck Surg..

[2] Scarpa A., Ralli M., De Luca P., Gioacchini F.M., Cavaliere M., Re M., Cassandro E., Cassandro C. (2021). Inner Ear Disorders in SCUBA Divers: A Review. J. Int. Adv. Otol..

[3] Stieler O., Loba W., Gawęcki W., Urbaniak-Olejnik M., Majewska A., Warchoł W., Hojan-Jezierska D. (2021). The impact of regular diving on the condition of the middle ear. Int. J. Occup. Med. Environ. Health.

[4] Smart D. (2024). Five consecutive cases of sensorineural hearing loss associated with inner ear barotrauma due to diving, successfully treated with hyperbaric oxygen. Diving Hyperb. Med..

[5] Lindfors O.H., Raisanen-Sokolowski A.K., Hirvonen T.P., Sinkkonen S.T. (2021). Inner ear barotrauma and inner ear decompression sickness: A systematic review on differential diagnostics. Diving Hyperb. Med..

[6] Mitchell S.J. (2024). Decompression illness—A comprehensive overview. Diving Hyperb. Med..

[7] Gempp E., Louge P., de Maistre S., Morvan J.B., Vallee N., Blatteau J.E. (2016). Initial Severity Scoring and Residual Deficit in Scuba Divers with Inner Ear Decompression Sickness. Aerosp. Med. Hum. Perform..

[8] Blatteau J.-E., Gempp E. (2025). Fit for diving after musculoskeletal decompression sickness- how to detect and manage bone lesions?. Undersea Hyperb. Med..

[9] Aldè M., Cantarella G., Piatti G., Ambrosetti U. (2023). Sudden hearing loss and early hyperbaric oxygen therapy—A preliminary study. Undersea Hyperb. Med..

[10] Alter I.L., Hamiter M., Han J., Leu C.S., Usseglio J., Lalwani A.K. (2025). Hyperbaric Oxygen and Sudden Sensorineural Hearing Loss: A Systematic Review and Meta-Analysis. Laryngoscope.

[11] Bagli B.S. (2020). Clinical efficacy of hyperbaric oxygen therapy on idiopathic sudden sensorineural hearing loss. Undersea Hyperb. Med..

[12] Joshua T.G., Ayub A., Wijesinghe P., Nunez D.A. (2022). Hyperbaric Oxygen Therapy for Patients with Sudden Sensorineural Hearing Loss: A Systematic Review and Meta-analysis. JAMA Otolaryngol. Head Neck Surg..

[13] Chin C.S., Lee T.Y., Chen Y.W., Wu M.F. (2022). Idiopathic Sudden Sensorineural Hearing Loss: Is Hyperbaric Oxygen Treatment the Sooner and Longer, the Better?. J. Pers. Med..

[14] Laupland B.R., Laupland K.B., Thistlethwaite K. (2024). Hyperbaric oxygen therapy for idiopathic sudden sensorineural hearing loss: A cohort study of 10 versus more than 10 treatments. Diving Hyperb. Med..

[15] Kim H., Kong S.K., Kim J., Lee H.M., Choi S.W., Lee I.W., Oh S.J. (2023). The Optimized Protocol of Hyperbaric Oxygen Therapy for Sudden Sensorineural Hearing Loss. Laryngoscope.

[16] Mason J.S., Buzzacott P., Gawthrope I.C., Banham N.D. (2023). A retrospective review of divers treated for inner ear decompression sickness at Fiona Stanley Hospital hyperbaric medicine unit 2014–2020. Diving Hyperb. Med..

[17] Van Der Wal A.W., Van Ooij P.J., De Ru J.A. (2016). Hyperbaric oxygen therapy for sudden sensorineural hearing loss in divers. J. Laryngol. Otol..

[18] Doolette D.J., Mitchell S.J. (2022). Extended lifetimes of bubbles at hyperbaric pressure may contribute to inner ear decompression sickness during saturation diving. J. Appl. Physiol..

[19] Parsons D., Utz E., Kidd G., Virgilio G. (2024). Inner ear decompression sickness after a routine dive and recompression chamber drill. Undersea Hyperb. Med..

[20] Sames C., Gorman D.F., Mitchell S.J., Zhou L. (2019). The impact of diving on hearing: A 10–25 year audit of New Zealand professional divers. Diving Hyperb. Med..

[21] Cavaliere M., De Luca P., Scarpa A., Strzalkowski A.M., Ralli M., Calvanese M., Savignano L., Viola P., Cassandro C., Chiarella G. (2022). Combination of Hyperbaric Oxygen Therapy and Oral Steroids for the Treatment of Sudden Sensorineural Hearing Loss: Early or Late?. Medicina.

[22] Moon R.E., Mitchell S.J. (2023). Hyperbaric Treatment of Air or Gas Embolism-Current Recommendations. Undersea Hyperb. Med..

[23] Stokes R.J., Watts D., Smerdon G., Hall S.D., Bunn L., Marsden J. (2025). Vestibular rehabilitation and recovery in divers with inner ear decompression sickness: A case series. Diving Hyperb. Med..

